# Peptide Platform as a Powerful Tool in the Fight against COVID-19

**DOI:** 10.3390/v13081667

**Published:** 2021-08-23

**Authors:** Michela Murdocca, Gennaro Citro, Isabella Romeo, Antonio Lupia, Shane Miersch, Bruno Amadio, Alessia Bonomo, Antonio Rossi, Sachdev S. Sidhu, Pier Paolo Pandolfi, Stefano Alcaro, Federica Carla Sangiuolo, Giuseppe Novelli

**Affiliations:** 1 Department of Biomedicine and Prevention, University of Rome Tor Vergata, Via Montpellier 1, 00133 Rome, Italy; miky.murdi@hotmail.it (M.M.); gennaro.citro46@gmail.com (G.C.); ale96.b@libero.it (A.B.); sangiuolo@med.uniroma2.it (F.C.S.); 2 Department of Health Science, University ‘Magna Graecia’ of Catanzaro, Campus “S. Venuta”, Viale Europa, 88100 Catanzaro, Italy; isabella.romeo@unicz.it (I.R.); alcaro@unicz.it (S.A.); 3 Net4Science Academic Spin-Off, University ‘Magna Graecia’ of Catanzaro, Campus “S. Venuta”, Viale Europa, 88100 Catanzaro, Italy; lupia@unicz.it; 4 The Donnelly Centre for Cellular and Biomolecular Research, University of Toronto, Toronto, ON M5S 3E1, Canada; shanemiersch@gmail.com (S.M.); sachdev.sidhu@utoronto.ca (S.S.S.); 5 Department of Molecular Genetics, University of Toronto, Toronto, ON M5S 3E1, Canada; 6 SAFU-IRE, Regina Elena Cancer Institute, 00128, Rome, Italy; Bruno.amadio@ifo.gov.it; 7 Institute of Translational Pharmacology, CNR, 00133 Rome, Italy; antonio.rossi@ift.cnr.it; 8 Renown Institute for Cancer, Nevada System of Higher Education, Reno, NV 89502, USA; ppandolf988@gmail.com; 9 IRCCS Neuromed, 86077 Pozzilli (IS), Italy; 10 Department of Pharmacology, School of Medicine, University of Nevada, Reno, NV 89557, USA

**Keywords:** COVID-19, peptides, protein–protein docking, conserved RBD region, SARS-CoV-2 variants, VSVpp.SARS-2S, drug design

## Abstract

Severe acute respiratory syndrome coronavirus 2 (SARS-CoV-2) has resulted in a global pandemic causing over 195 million infections and more than 4 million fatalities as of July 2021.To date, it has been demonstrated that a number of mutations in the spike glycoprotein (S protein) of SARS-CoV-2 variants of concern abrogate or reduce the neutralization potency of several therapeutic antibodies and vaccine-elicited antibodies. Therefore, the development of additional vaccine platforms with improved supply and logistic profile remains a pressing need. In this work, we have validated the applicability of a peptide-based strategy focused on a preventive as well as a therapeutic purpose. On the basis of the involvement of the dipeptidyl peptidase 4 (DPP4), in addition to the angiotensin converting enzyme 2 (ACE2) receptor in the mechanism of virus entry, we analyzed peptides bearing DPP4 sequences by protein–protein docking and assessed their ability to block pseudovirus infection in vitro. In parallel, we have selected and synthetized peptide sequences located within the highly conserved receptor-binding domain (RBD) of the S protein, and we found that RBD-based vaccines could better promote elicitation of high titers of neutralizing antibodies specific against the regions of interest, as confirmed by immunoinformatic methodologies and in vivo studies. These findings unveil a key antigenic site targeted by broadly neutralizing antibodies and pave the way to the design of pan-coronavirus vaccines.

## 1. Introduction

Severe acute respiratory syndrome coronavirus 2 (SARS-CoV-2) causes severe respiratory syndrome (COVID-19) and represents a global health threat. This pathology is characterized by high mortality due to a direct cytotoxic viral effect and severe systemic inflammation [1]. 

Coronaviruses tropism is primarily determined by the ability of the spike entry glycoprotein (S protein) to bind to a cell surface receptor. The S protein is divided into two subunits: S1, which includes the receptor-binding domain (RBD), and S2 [2]. Recent studies revealed that the first step of SARS-CoV-2 entry into cells of the respiratory tract depends on binding to the receptor angiotensin-converting enzyme 2 (ACE2) but also the membrane protein dipeptidyl peptidase 4 (DPP4) membrane protein [3]. Moreover, DPP4, also known as CD26, is as relevant as ACE2 in the pathogenesis of virus entry [4]. In fact, many strategies to prevent the virus from entering the cell are based on interfering with RBD through the design and de novo synthesis of peptides belonging to the interacting site of the cell-membrane receptors. Although at relatively early stages, these studies evidence the potential of computational tools for developing novel strategies against the COVID-19 pandemic. 

The S protein of SARS-CoV-2 is the main antigen to generate neutralizing antibodies in mammals. To date, several different recombinant neutralizing antibodies have been allowed emergency use authorization (EUA) for COVID-19 treatment [5,6,7]. 

In addition, mRNA and vector-based vaccines encoding the protein derived from SARS-CoV-2 isolated early in the pandemic from Wuhan, China, have been used. To date, four vaccines have already been approved by European and American authorities for preventing COVID-19, but the development of additional vaccine platforms with improved supply and logistics profiles remains a pressing need. Moreover, there are no specific antiviral drugs available for COVID-19 treatment; thus, a therapeutic targeted to directly inhibit SARS-CoV-2 is needed urgently [8].

After the detection of the first cases in Wuhan, the D614G change within the genetic material of the S protein became dominant early in the pandemic, being associated with increased transmissibility [9,10]. Recently, many SARS-CoV-2 variants have emerged, which seem to show increased transmissibility due to mutations within the S protein. Among these variants, some of them are of clinical and diagnostic interest such as the B.1.1.7 (Alpha or UK variant), which has been associated with a wave of COVID 19 cases [10,11,12], the variant B.1.351 also termed the South Africa (Beta) variant [13], and P.1 also called the Brazil variant or Gamma [14]. Although enormous expenditures have been made toward the rapid development of a vaccine, there are no certainties that vaccines against these coronavirus strains will supply long-lasting effective protection [15,16,17]. 

Peptide-based inhibitors represent an interesting therapeutic approach ensuring efficiency, specificity, and tolerability [18,19]. Peptides are ideally suited to mimic natural ligands and thereby function in an antagonistic or agonistic manner. Furthermore, they are able to physiologically disrupt functional complexes due to their small size and specific binding properties. Proteins form homo- or heteromeric (macro)molecular complexes and intricate networks by interacting with small molecules, peptides, nucleic acids, or other proteins. Many protein–protein interactions are mediated by hot-spots, which comprise only a small part of the large binding interface but account for a large fraction of the binding energy. Thus, these hot-spots provide an ‘Achilles heel’ for pharmaceutical interventions aimed at the disruption of functional protein–protein complexes. Thus, deciphering and characterizing peptide–protein recognition mechanisms is central for the development of peptide-based strategies to interfere with endogenous protein interactions or improvement of the binding affinity and specificity of existing approaches. Importantly, a variety of computation-aided rational designs for peptide therapeutics have been developed, which aim to deliver comprehensive docking for peptide–protein interaction interfaces [20]. Once selected, these peptides can be optimized for their binding affinity using peptide arrays. Their chemical composition makes peptides highly specific and versatile: they can be easily modified by both shortening their sequence, changing amino acids, or adding parts (some dextrorotatory amino acids) for increasing their half-life. Their stabilization can be achieved by the introduction of non-natural amino acids to form so-called peptidomimetics that are resistant to cellular proteases. Moreover, lipocalins and peptide aptamers represent scaffolded binding structures with unique binding characteristics and enhanced stability. In case of extracellular targets, such as cell surface receptors or pathogens in patients’ plasma, peptide inhibitors have direct access. In recent years, peptides have represented promising candidates for targeting challenging binding interfaces with satisfactory binding affinity and specificity. Specifically, antiviral peptides conventionally target structures essential for viral attachment, fusion, replication, transcription, and maturation. 

On the other hand, the peptide platform can be employed as a preventive approach formulated with synthetic peptides that allow focusing the response specifically versus selected epitopes. This approach guarantees the stimulation of the host immune system by producing specific neutralizing antibodies against selected sequences that cannot represent immunodominant sites using the whole protein as antigen. Data reported by He et al. [21] demonstrate that neutralizing antigenic linear epitopes are present in the RBD of the S protein, despite the absence of algorithmic identifiable immunodominant sites. These data are relevant considering that often, even in the same antigen, the dominant sites restrict the immune response and thus reduce the efficacy of the antiserum [22,23,24].

- In this paper, we have demonstrated the applicability of a peptide-based strategy, focused both to a preventive as well as a therapeutic purpose.
- Specifically, we have selected and synthetized peptide sequences located within the RBD conserved region [25]. Four 25-residue sequences (comprising amino-acids 322–508), were predicted as highly potential antiviral peptides (T-cell epitopes) to impede the pathogenic process of SARS-CoV-2 through immunoinformatics methodologies. Then, we administered the peptides in vivo in mice in order to stimulate the production of IgG antibodies specific against the regions of interest, and sera have been tested to evaluate their neutralizing activity.
- In parallel, based on the established involvement of the DPP4 receptor, a computational approach helped to rationalize the ability of two peptides to bind the S protein by carrying out a protein–protein docking analysis in order to investigate the binding affinity for RBD. Afterwards, the peptides bearing DPP4 sequences have been produced and used in vitro to assess their capacity to block the VSV* ∆G-Fluc pseudovirus infection.

## 2. Materials and Methods

### 2.1. Peptide Design and Production

ACE2 and DPP4 peptides were designed evaluating those reported in Nianshuang Wang et al. [26]. Instead, the sequences of the four peptides of spike were designed by us, choosing the area comprising the cryptic domain on the RBD sequence (Table 1).

### 2.2. Protein Preparation

The cryo-EM structures of the SARS-CoV-2 S protein in a partially open state (PDB ID: 6VSB) [27] was adopted for the computational study. Fully glycosylated and structurally complete (missing residues rectified) S protein head-only models, including residue 1–1146, was obtained from the CHARMM-GUI COVID archive [28]. Then, protein and glycans were solvated in a cubic periodic box with water extending 2 Å outside the protein in all sides using a TIP3P water model [29] and CHARMM36m [30] as a force field. The system was maintained at a salt concentration of 0.15 M by adding appropriate Na^+^ and Cl^−^ counter ions to neutralize the system to maintain the physiological condition. Then, the systems were submitted to 50,000 steps of the steepest descent algorithm to minimize the energy under solvation conditions. 

### 2.3. Preparation of Peptides and Molecular Dynamic Simulations (MDs)

Starting from the ACE2-RBD crystallographic model (PDB: 7KMB) and taking into account their protein–protein interactions, the helical peptide sequence (21–43) was first extrapolated from the α1 helix of ACE2 and subsequently used as a starting point to present 100 ns of MD [31]. Regarding DPP4_270–295_ and DPP4_318–343_ preparation, the amino acid sequences (residues 270–295) and (residues 318–343) derived from the X-ray crystallographic structure of DPP4 (PDB code: 2G63) [32] were extracted and simulated for 100 ns to catch the conformational variability of the investigated peptides. MD simulation of the peptides was performed through GROMACS 5.1.4 [33], by using CHARMM27 [34] as the force field and TIP3P as the water model, thus solvating the structure of each peptide in a cubic periodic box with water extending 2 Å outside the peptide. The systems were maintained at an appropriate salt concentration of 0.15 M by adding 1 Na^+^, 3 Na^+^, and 4 Na^+^ counter ions for DPP4_270–295_, DPP4_318–343_, and ACE2_21–43_, respectively. The systems use the steepest descent algorithm to minimize the energy by 50,000 steps under solvation conditions. Subsequently, the V-type heavy-scale thermostat with a coupling constant of 0.1 ps was used to gradually heat the system to reach 310 K temperature to balance in the NVT ensemble. Then, a Parrinello Rahman constant with a coupling constant of 0.1 ps was used to maintain the solvent density at 1 bar and 310 K, and the equilibrium was carried out in the NPT ensemble. The LINCS algorithm was adopted to constrain all H-bonds during the equilibration, while long-range ionic interactions were approximated using the particle-mesh Ewald (PME) algorithm. Finally, 100 ns (50 million time steps) unconstrained MDs were carried out for each peptide at the equilibrium state, and the GROMACS package was used to analyze the results. Cluster analysis of the resultant trajectories was performed using the method described by Daura et al. [35] using the gmx cluster built-in module of Gromacs. GROMOS, a Root Main Square Deviations (RMSD) conformational clustering algorithm, was used to extract the maximally occupied clusters by taking into account the peptide conformation with the lowermost RMSD to the centroid, for a total of three representative structures for each peptide.

### 2.4. Protein–Protein Binding Affinity Prediction

A flexible protein–protein docking tool, HADDOCK 2.4, was used for docking simulations [36]. This tool combines Coulomb electrostatic energies, non-bonded intermolecular Van der Waals, and empirically derived desolvation energies and buried surface area. The spike glycoprotein amino acids exposed to the surface (K417, K458, N487, Y489, Q493, G496, T500, G502) and all amino acids of the peptides were considered as active residues. For each representative structure of the two peptides, the sampling parameters were as follows: 10,000 structures for rigid-body docking, 400 structures for the final refinement, and a cut-off equal to 5.0 to define neighboring flexible regions. According to the 

HADDOCK score and the docking RMSD value, the obtained complexes of DPP4_270–295_ and DPP4_318–343_ with RBD were analyzed for binding affinity ΔG (kcal mol ^−1^). In order to validate the docking protocol, we also performed the molecular recognition of both ACE2 and the truncated portion ACE2_21–43_ to the RBD domain. To better predict the binding affinity and stability based on structural properties of the investigated protein–protein complexes, the best cluster of DPP4_270–295_ and DPP4_318–343_ in complex with S protein were submitted to the protein binding energy prediction (PRODIGY) server [37,38]. The stability of the protein–protein complex was measured through the dissociation constant K_d_ (M) at 37 °C. Schrodinger’s Maestro visualization program [39] was operated to visualize and generate all the structures under investigation.

### 2.5. T-Cell Epitope Identification

The investigated spike peptides sequences (RBD_484–508_, RBD_453–476_, RBD_402–427_, and RBD_322–341_) were submitted to MHCI-binding prediction using the IEDB analysis resource CombLib tool [40] applying Scoring Matrices derived from the Combinatorial Peptide Libraries (Comblib_Sidney2008) method. T-cell epitope lengths were defined as 9 mer and H2-Dd, H2-Kd, and H2-Ld alleles were included for BALB/c MHC class I. 

The peptides that were predicted to bind to MHC class I with percentile rank  ≤  1 were considered epitopic sequences.

The NetCTL 1.2 server was also used for the identification of the T-cell epitope [41]. The prediction method integrated peptide MHC-I binding, proteasomal C terminal cleavage, and TAP transport efficiency. The epitope prediction was restricted to 12 MHC-I supertypes. MHC-I binding and proteasomal cleavage were performed through artificial neural networks, and the weight matrix was used for TAP transport efficiency. The parameter we used for this analysis was set at a threshold of 0.7. A combined algorithm of MHC-I binding, TAP transport efficiency, and proteasomal cleavage efficiency was selected to predict overall scores [42,43]. 

### 2.6. Cells

The African green monkey kidney Vero E6 cell line (kindly gifted by Spallanzani Institute, Rome, Italy) was maintained in Dulbecco’s Modified Eagle Medium (DMEM; Gibco, Thermo Fisher Scientific, Waltham, MA, USA) supplemented with 10% fetal bovine serum (FBS; Gibco, Thermo Fisher Scientific, Waltham, MA, USA) at 37 °C in a humidified atmosphere of 5% CO_2_.

### 2.7. Preparation of Pseudotyped Particles

For the generation of SARS-CoV-2 S-pseudotyped particles, we used a replication-deficient VSV vector that lacks the genetic information for VSV-G (VSV*DG-fLuc) (a kind gift from Hoffmann lab, German Primate Center–Leibniz Institute for Primate Research, Gottingen, Germany) [10]. VSV*DG-fLuc was propagated on mefepristone-induced BHK-G43 cells, and the infectious titers were calculated as fluorescence-forming units per milliliter (ffu/mL).

293T cells were transfected with the SARS-CoV-2-S plasmids expressing the WT (B.1 lineage) protein and the variants D614G, United Kingdom (UK, B.1.1.7 lineage), South Africa (SA, B.1.351 lineage), and Brazil (BR, P.1 lineage) SARS-CoV-2 spikes (a kind gift from Hoffmann lab, German Primate Center–Leibniz Institute for Primate Research, Gottingen, Germany). At 24 h after transfection, cells were infected with VSV*DG-fLuc (m.o.i. of 3 ffu/cell). After incubation at 37 °C for 1 h, virus inocula were removed, and monolayers were washed twice with DMEM and incubated with DMEM containing 5% FCS and supplemented with anti-VSV-G antibodies (I1, mouse hybridoma supernatant from CRL-2700;) in order to neutralize residual input virus. Pseudotyped particles were harvested at 24 h post infection, clarified from cellular debris by centrifugation, and stored at –80 °C until use. 

### 2.8. Serum Anti-Peptide Production

The immunization regimen employed did not induce detectable pathological effects on treated animals (weight loss, loss of appetite, reduced mobility) either during or at the end of vaccinations.

After collecting a pre-immunization bleed, SPF BALB/c mice were immunized with 100 µg of peptide formulate in 50 µL PBS and 50 µL Freund’s Adjuvant complete three times intramuscularly with an interval of one week. After 20 days from the last immunization, a boost (200 µg in 50 µL) was injected intramuscularly. Sera were collected at 5 and 10 days after the last injection. Plasma from immunized animals was heat inactivated at 56 °C for 1 h and then stored at 4 °C until use. Whole antiserum from treated animals were tested to react with S protein in an ELISA test.

All procedures were in accordance with institutional guidelines under the control of the Italian Ministry of Public Health (Italian Law D.lgs 26/2014) and conform to Directive 2010/63/EU and with the Guide for the Care and Use of Laboratory Animals. 

### 2.9. Determination of Anti-SARS-CoV-2 S-Protein RBD IgG Antibody Circulating Levels

Animals were divided in 4 groups, and blood was collected with a Hamilton syringe. Blood samples were incubated 1 h at room temperature to allow the formation of blood clot. Following centrifugation (15 min, 2,000 g), mouse sera were recovered and stored at –80 °C. Mouse IgG antibody levels were assessed from animal sera with anti-SARS-CoV-2 IgG Antibody to S-protein RBD by solid-phase enzyme labeled chemiluminescent immunometric assay. 

The mice cohorts were assigned randomly to receive S-protein peptides (200 µg/mL) or vehicle via intramuscular injection. The vehicle groups received equal volumes of saline solution. The total injected volumes were 50 μL. 

### 2.10. In Vitro Neutralization Assay of DPP4 Peptide

For neutralization experiments with SARS-CoV-2 VSV-based pseudoparticles, Vero- E6 cells were seeded in 96-well plates in 100 μL minimal essential medium Eagle at 30,000 cells/well. The next day, diluted peptide DPP4 in PBS was added to SARS-CoV-2 VSV-based pseudoparticles CoV2S-PPs at 1:1 dilution with a maximum of 2% serum. DPP4/pseudovirus mixtures were incubated for 1 h at 37 °C and then added to Vero E6-cells in triplicates, and cells were incubated at 37 °C and 5% CO_2_. Then, 48 h post transduction, Firefly luciferase activity was measured using the Promega Luciferase Assay System (E1501). 

### 2.11. Quantification and Statistical Analysis

All the experiments were performed in technical duplicates, and data were analyzed using GraphPad Prism 8 and the SPSS program, version 25 (IBM Corp, Armonk, NY, USA). 

## 3. Results

### 3.1. Structural Comparison among DPP4 Peptides Docked into RBD Binding Pocket

**Figure 1:**
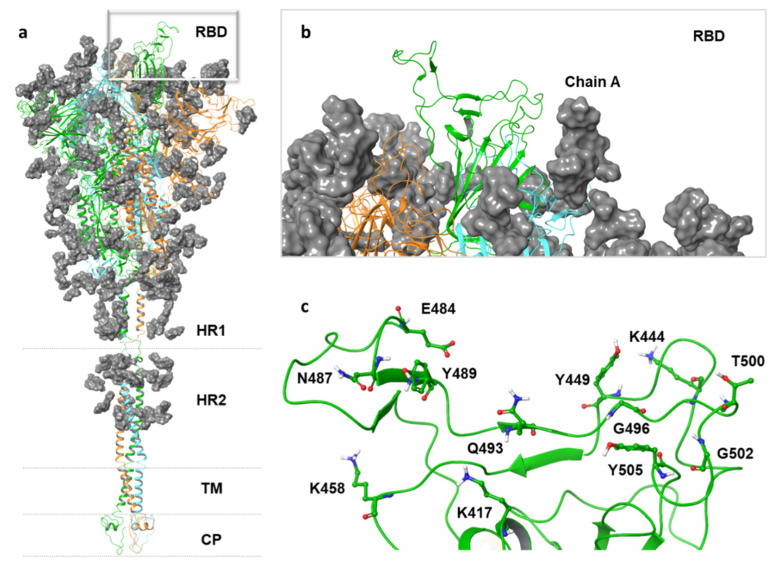
(**a**) A model structure of full-length SARS-CoV-2 S protein is shown in the left panel. All chains (A,B,C) are represented by a secondary structure in cartoon representation, while the gray surface represents all the glycans. (**b**) The focus on chain A is depicted with green cartoons in the top right panel. (**c**) The spike glycoprotein amino acids exposed to the surface (K417, K458, N487, Y489, Q493, G496, T500, and G502) are shown as ball and stick representations, based on atom type model, in the bottom right panel.

Starting from the pre-fusion trimeric Sprotein structure (PDB code: 6VSD), we chose to use the open state of the S protein because it displays active conformation with one RBD up and ready for ACE2 binding. The adopted structure comprised all potential glycans attached to the S-protein surface due to their role as shielding devices for immune evasion but also as pivotal elements for virus infectivity [44]. Indeed, the S protein placed on the membrane of SARS-CoV-2 promotes viral entry into host cells when its RBD, which includes residues 319–541, interacts with the human ACE2 receptor. Among those, the critical contacting elements that engage binding interactions with ACE2 are termed the Receptor Binding Motif (RBM), consisting of residues 437–508 [27]. Our initial structure contains a heptad repeat 1 and 2 (HR1 and HR2), trans-membrane (TM), and cytoplasmic (CP) domain provided from pre-built simulation systems collected into the COVID-19 Archive in CHARMM-GUI (Figure 1a). After structure minimization, we focused our attention on chain A due to its essential role in the direct binding to the human ACE2 receptor (Figure 1b,c) for the further docking simulations. 

**Figure 2:**
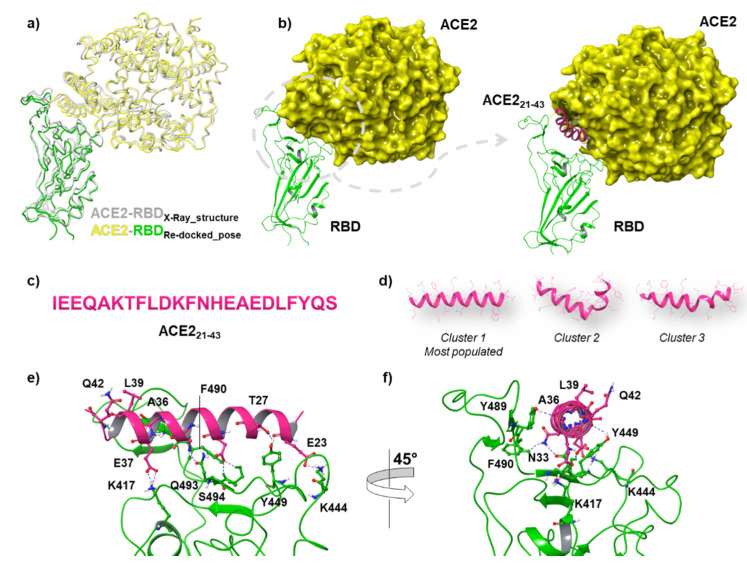
(**a**) Superposition between the X-ray structure of ACE2 in complex with RBD and the best re-docked pose after docking simulations through the HADDOCK tool. (**b**) Right-hand images show zoomed-in context of the ACE2_21–43_ peptide at the interface between ACE2 and RBD. (**c**) Linear sequence of a truncated portion (21 to 43 aa) of the ACE2 receptor. (**d**) Three-dimensional (3D) representation of the three representative clusters of ACE2_21–43_, the first of which is the most populated. Key contacting elements inside (**e**) front view of the RBD-up/ACE2_21–43_ conformation, and (**f**) side view of the RBD-up/ACE2_21–43_. All amino acid residues involved in H-bonds (dark blue) and salt bridges (purple) are shown in stick representation.

Considering that the binding residues for ACE2 comprise a wide protein-binding pocket on the RBD portion, it may not be easy to identify a small peptide that is able to compete with the entire RBD. In this respect, the helical peptide sequence of ACE2_21–43_ consisting of 23 amino acids (Figure 2c) resulted in binding to RBD with low nanomolar affinity [45], thus encouraging the efforts for targeting the RBD with new designed peptides. Our docking protocol was first validated by docking ACE2 into the RBD binding site. The Root Mean Square Deviation (RMSD) value between the co-crystallized structure of ACE2-RBD (PDB code:7KMB) and the related best re-docked conformation was found to be 1.33 Å, thus revealing the reliability of the docking protocol (Figure 2a).

Afterwards, we also tested the behavior of ACE2_21–43_ docking RBD pocket (Figure 2b). To do this, we carried out 100 ns of MDs for both ACE2_21–43_ and the investigated DPP4 peptides, such as DPP4_270–295_ and DPP4_318–343_, in order to capture their conformational variability. Analyzing MDs trajectory, we observed that ACE2_21–43_ remained in stable conformation during the whole simulation, resulting in an average RMSD value of 0.28 Å. After clustering trajectory, three representative structures for the peptide were used for further molecular docking recognition (Figure 2d). By using the HADDOCK 2.4 tool, docking analysis revealed that the ACE_21–43_ structure established remarkable H-bonding interactions with the RBD domain (HADDOCK score equal to −89). In particular, E37, A36, and E23 were able to form the H-bonds and salt bridges with K417, Y489, and K444. Moreover, T27, N33, and D30 interacted with Y449, F490, Q493, and S494 of the RBD loop, respectively (Figure 2e,f), with a total Buried Surface Area (BSA) of 1221.5 +/− 41.4. At this point, we applied the same MDs procedure for DPP4_270–295,_ and DPP4_318–343_ peptides, and the three representative structures for each peptide were docked into the RBD of the S protein. Our computational results revealed that DPP4_270–295_ showed a lower HADDOCK score compared to DPP4_318–343_ (Table 2), suggesting the higher affinity of DPP4_270–295_ for RBD.

**Figure 3:**
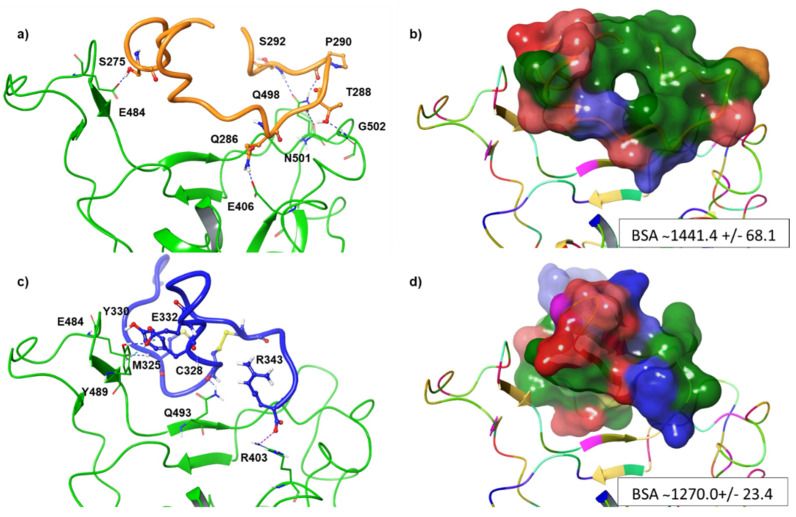
Key contacting elements inside (**a**,**b**) the RBD-up/DPP4_270–295_ conformation, and (**c**,**d**) the RBD-up/DPP4_318–343_. Panels (**a**) and (**c**) show in stick representation all side chains involved in H-bonds (dark blue), π–π interactions (cyan), and salt bridges (purple). Panels (**b**) and (**d**) show the BSA area of RBD-up/DPP4_270–295_ complex and RBD-up/DPP4_318–343_, respectively. The surface area of the peptides is shown in solid style and coloured according to the atom type.

Concerning DPP4_270–295_ in complex with RBD, it was observed that the pivotal interactions involved the T470-F490 loop and Q498-Y505 residues within RBD. In particular, the most representative H-bonding interactions were highlighted among E406, E484, Q498, N501, and G502 of RBD, and Q286, S275, S292, P290, and T288, of DPP4_270–295_ (Figure 3a). DPP4_318–343_ was accommodated into the RBD cavity through a hydrogen bond and a π–π interaction between Y489 of RBD and E332 or Y330 of DPP4_318–343_, respectively. M325 and C328 of DPP4_318–343_ also made two H-bonds with E484 or Q493, respectively, while a salt bridge was observed between R343 of DPP4_318–343_ and R403 of RBD (Figure 3c). The most relevant VdW contribution was the driving force of the favored binding of DPP4_270–295_ in complex to RBD in comparison to that of DPP4_318–343_ due to their more extended BSA equal to 1441.4 +/− 68.1 Å2 (Figure 3b). Indeed, the strength of binding between two proteins is dependent on interface size [46] and thus its corresponding interface area [47,48], as shown in Figure 3b,d. VdW forces, though still weaker than H-bonding, hydrophobic attraction, and ionic interaction, play a key role in the stabilization of protein–protein complexes besides the corresponding BSA [49]. This is also considering that the binding affinity of the protein–protein complex is also related to dissociation constants (K_D_), pH, and temperature [50]. The best clusters of DPP4_270–295_ and DPP4_318–343_ in complex to RBD were submitted to PRODIGY server [37]. DPP4_270–295_ complexed to RBD showed a higher binding affinity value (−11.8 kcal mol^−1^) with respect to the DPP4_318–343_ complex, which resulted in a ΔG binding affinity value equal to −9.0 kcal mol ^−1^ at 37  °C (Table 3). Likewise, the predicted dissociation constant (K_D_) of DPP4_270–295_ was smaller than that of DPP4_318–34,_ thus indicating its strong binding affinity and high stability with RBD. 

### 3.2. DPP4 Peptides Block SARS-CoV-2 Entry into Cells

**Figure 4:**
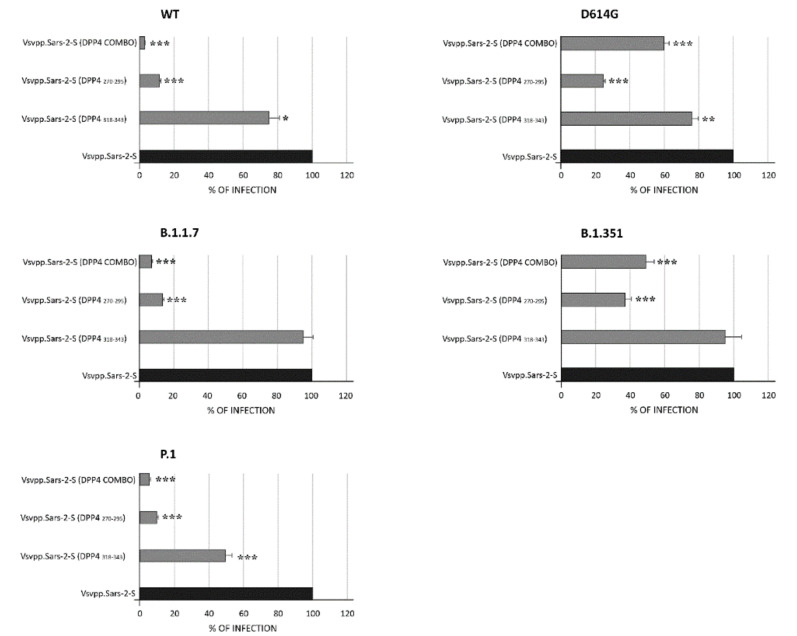
Neutralizing assay by DPP4 peptides. VeroE6 cells were inoculated with pseudotypes particles bearing the S proteins of the indicated Vsvpp.SARS-2-S WT or variants (i.e., D614G,B.1.1.7,P1,B.1.351), together with DPP4 _270–295_, DPP4 _318–349_, or a combination of the two. Trasduction efficiency was quantified by measuring virus encoded luciferase activity in cell lysates at 48 h post transduction and expressed as percentage. Data presented are the average from three biological replicates. Error bars indicate the standard deviation ±SD. * *p* < 0.05, ** *p* < 0.01, *** *p* < 0.001.

Successively, for testing the efficiency of DPP4 peptides (specifically DPP4_270–295_ and DPP4_318–343)_, for inhibition of the entry driven by WT and variant S proteins (i.e., D614G, B1.1.7, B1.351, and P1), pseudoviruses carrying either SARS-CoV-2 WT or those variants were used for the infection (Figure 4). For pseudotyping, we used a replication-deficient VSV vector that lacks the genetic information for VSV-G and instead codes for two reporter proteins, enhanced green florescent protein (GFP) and firefly luciferase (Fluc), VSV*∆G-Fluc. This system accurately mimics key aspects of SARS-CoV-2 entry into cells [10]. After incubating a mixture of peptide/virus for 1 h at 37 °C, VeroE6 cells were infected for 48 h. Successively, the efficiency of virus entry was first observed by evaluating GFP fluorescence (data not shown) and after was specifically quantified by performing luciferase assay. Each value was normalized with respect to Vsvpp.Sars-2-S alone (Figure 4). As observed in Figure 4, the DPP4_318–343_ peptide did not decrease the virus capacity to enter into target cells except for WT, D614G, and P.1 pseudotypes, for which 75%, 76%, and 49.4% (*p* < 0.05, *p* < 0.01, *p* < 0.001) of virus infection were observed. On the contrary, the DPP4_270–295_ peptide showed a higher capacity of inhibition for WT and its variant particles, going from 9.8% (P.1) to 37% (B.1.351) (*p* < 0.001). When used in combination, the effect appeared synergistic in all cases (*p* < 0.001). 

Lastly, we examined the theoretical binding affinity of DPP4_270–295_ peptide for the variant S proteins that contain mutations in the RBD, such as B1.1.7, B1.351, and P.1. Docking simulations revealed that in the presence of K417T, E484K, and N501Y for the P.1 variant, DPP4_270–295_ was able to recognize the RBD with a higher HADDOCK score value equal to −95.3, with respect to the other two analyzed variant S proteins. Indeed, in the presence of K417N, E484K, and N501Y for B1.351 or N501Y for B1.1.7, the HADDOCK score of DPP4_270–295_ was −89.5 and −84.3, respectively (Table 4). Notably, in the P.1. variant, DPP4_270–295_ established several interactions with R403, C480, F486, Q493, Y489, S494, and V503 of the RBD. On the other hand, in B1.351, it was found that only four residues of DPP4_270–295_ such as D274, S275, L276, and P290 of DPP4_270–295_ were involved in binding with N487, Y489, and two mutated amino acids K484 and Y501, thus rationalizing its less favored theoretical binding affinity to RBD.

### 3.3. T Cell Epitope Identification 

**Figure 5:**
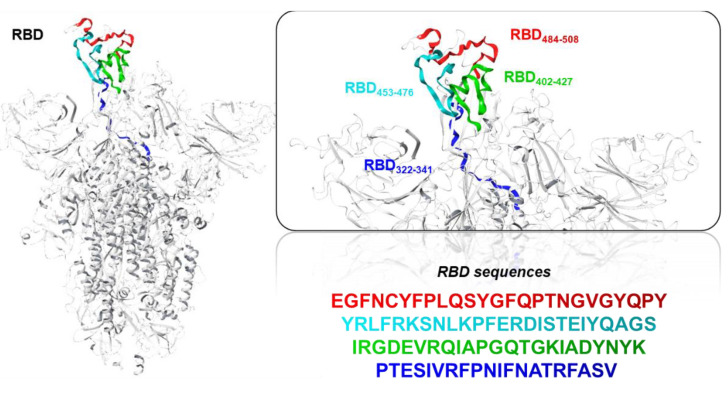
Illustration of the RBD S-protein main chain as a gray carbon cartoon. Right-hand images show zoomed-in context of the four sequences inducing RBD-specific antibodies, as proposed in the present study, which were coloured as red (RBD_484–508_), cyan (RBD_453–476_), green (RBD_402–427_), or blue (RBD_322–341_) main chain ribbons.

Four truncated portions of the S protein (Section 2.1), shown in Figure 5, were subjected to the IEDB MHC I binding prediction tool, applying 9-mer lengths coverage of T-cell epitopes. 

According to the generated data reported in Table 5, a lower score was attributed to YFPLQSYGF and EGFNCYFPL peptides, resulting in a percentile rank of 0.8. These sequences are part of the RBD_484–508_ (peptide 1), thus representing the promising candidate for further investigation on the identification of epitopes derived from the SARS-CoV-2 spike glycoprotein.

The NetCTL server was used to predict the potent T-cell epitopes from the four S protein–peptide sequences. Based on the high combinatorial score (Table 6), the best epitopes were predicted to be the ERDISTEIY and FQPTNGVGY sequences with a prediction score of 1.1686 and 0.7583, respectively, for the A1 supertype of MHC class I. Meanwhile, the FQPTNGVGY sequence exhibited the highest binding affinity of 0.2611, which was coupled with a relatively high score for proteasomal C-terminal cleavage and transport affinity for the A26 supertype, with a prediction score of 0.9848. These resulted sequences are located within the RBD_484–508_ (peptide 1) and RBD_453–476_ (peptide 2), thus assuming their involvement to trigger an efficacious immune response.

### 3.4. Murine Humoral Response and Neutralizing Activity of IgG Elicited by RBD Peptide

In light of bioinformatics analyses, we immunized SPF BALB/c mice with four 25-residue peptides belonging to different cryptic areas of the S protein not subject to mutations (four mice per group). Animals were treated with 100 µg of peptides for three times over with an interval of one week. Ten days after the last injection, the sera of treated animals were tested for reaction with S protein. Sera taken from mice treated with peptide RBD_484–508_ (peptide 1) and RBD_453–476_ (peptide 2) reacted with the whole S protein in an ELISA, while the other two peptides (RBD_402–427_: peptide 3 and RBD_322–341_: peptide 4) gave less relevant results. Specifically, a value of 341 ng/mL mouse IgG antibody levels were assessed in mice injected with peptide 1. An average of 42 ng/mL was measured for peptide 2 and 12.7 ng/mL and 13 ng/mL were measured for peptides 3 and 4, respectively. When a combination of all four peptides was used for injection, a value of 7.8 ng/mL was measured, which was probably due to an interaction among them. Importantly, an antibody titer boost was observed mainly in the mice group treated with the S protein peptide comprising the conserved region of the RBD_484–508_. 

**Figure 6:**
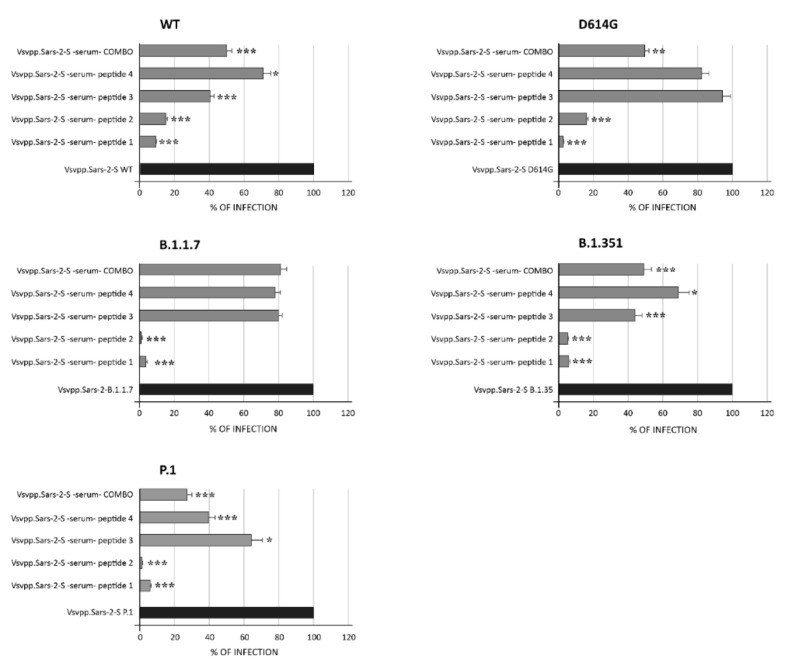
Neutralizing activity of IgG elicited by spike RBD peptides. VeroE6 cells were incubated with pseudotypes particles bearing the S proteins of the indicated Vsvpp.SARS-2-S WT or variants (i.e., D614G, B.1.1.7, P1, B.1.351) and together with serum containing antibodies elicited by spike peptides 1, 2, 3, or 4, or their combination. Transduction efficiency was quantified by measuring virus-encoded luciferase activity in cell lysates at 48 h post transduction and expressed as percentage. Data presented are the average from three biological replicates. Error bars indicate the standard deviation ±SD. * *p* < 0.05, ** *p* < 0.01, *** *p* < 0.001.

We next sought to analyze whether the RBD-specific antibodies induced by the four RBD peptides were able to neutralize SARS-CoV-2 WT and the variants D614G, B.1.1.7, B.1, B.1.351, and P.1 lineage. To this end, we initially used a pseudovirus-based neutralization assay with a luminescence readout. Then, the neutralizing capacity of peptide-induced antibodies was quantified by luciferase assay (Figure 6). 

As shown, a range of infection from 2.8 to 9.3% was assessed for peptide 1 in WT and variants (*p* < 0.001), while values ranging from 0.96 to 16.2% were assessed for peptide 2 in all pseudotype lineages tested (*p* < 0.001). When a combination of all four peptides was used, a range from 27.3 to 49% of infection was observed, demonstrating a discrete efficiency of infection inhibition. These results suggest that antibodies elicited by peptides corresponding to the conserved RBD are functional against the current most commonly circulating SARS-CoV-2 variants.

## 4. Discussion

The rational design of vaccines needs to be rapidly implemented to create the next generation of vaccines for the stimulation of optimal immune responses. One chance is represented by the use of epitope-specific based vaccines formulated with synthetic peptides, which allow focusing the response on the selected epitope (and not on the whole protein) that stimulates neutralizing antibodies. Nevertheless, peptides and small molecules that interfere with protein–protein interactions (PPIs) are in high demand as therapeutic agents in pharmaceutical industries due to their potential to modulate disease-associated protein interactions. These small molecules and inhibitory peptides are attractive drug candidates [51]. These strategies have turned peptide therapeutics into a leading industry with nearly 20 new peptide-based clinical trials annually. In fact, there are currently more than 400 peptide drugs that are under global clinical development with over 60 already approved for clinical use in the United States, Europe, and Japan. Accumulating evidence has suggested that better identification of targetable disease-associated PPIs and optimization of peptide drug binding characteristics will be key factors for their clinical success [51]. In comparison to small molecules, such as proteins and antibodies, peptides indeed represent a unique class of pharmaceutical compounds attributed to their distinct biochemical and therapeutic characteristics. 

In recent years, peptides with balanced conformational flexibility and binding affinity that are up to five times larger than small molecule drugs have attracted enormous attention. 

Peptide–protein interactions are ubiquitous in living cells and are an important part of the entire protein–protein interaction network [52]. It was found that peptide-mediated interactions are estimated to make up to 40% of all these interactions [53]. 

These interactions have attracted increasing attention due to their role in signaling and regulation and are therefore attractive targets for computational structure modeling [54]. Generally, to study peptide-mediated interactions, the structures of both receptor and peptide are needed [55]. Therefore, a structural database of peptide–protein interactions is valuable for not only the understanding of existing peptide–protein interplay but also the development of new docking algorithms for peptide drug discovery [56]. This means that synthetic peptides can be designed to change specific interactions, such as between DPP4 and the S protein, or other signaling pathways inducing in vivo humoral response. The present study identifies SARS-CoV-2 S-protein crypto antigenic epitopes and provides serological evidence of immunoresponses in mice, and it also offers initial useful information for the use of specific cross-reactive antigenic epitopes of S protein instead of the entire molecule in the design of a vaccine against different virus subtypes. This study is the first report regarding antiviral peptides with activity against all studied SARS-CoV-2 variants. 

Nowadays, by using immunoinformatics and computational studies, it is possible to identify epitopes in a protein for B cells and for T cells in order to generate vaccines. By using immunoinformatics methodologies, RBD_484–508_ and RBD_453–476_ were identified as highly promising antiviral peptides (T-cell epitopes) to impede the pathogenic process of SARS-CoV-2. Our data demonstrate that they are able to induce potent anti-SARS-CoV-2 neutralizing antibody responses (including the most common variants of concern) and confer significant protection upon SARS-CoV-2 challenge, as indicated by the in vitro assay regarding the percentage of infection and thus inhibition of virus host cell entry. 

Among the obstacles to overcome in the COVID-19 vaccination campaign is the reduction of the immunizing activity of current vaccines due to the appearance of variants in the S protein. It is likely that the host’s immune system is antigenically attracted to the areas of the S protein where the virus can then mutate without altering its biological activity. In fact, if there are no variants in some well-defined areas of the S protein, it means that either they do not occur or alternatively they do occur but are not compatible with viral reproduction. 

It is known that there is bias and immunodominance (ID) in the immune response, which sometimes reduces efficacy. The phenomenon of immunodominance often involves variations in immunogenicity even between different sites of the same antigen. On the other hand, it is known that in large antigens, this phenomenon also occurs as a function of individual polymorphisms [57,58,59,60]. 

Thus, this epitope strategy (small antigen to overcome immunodominant problems in the same protein) represents an excellent approach to be explored for a vaccine, considering also that selected epitopes are common to all SARS CoV-2 strains as deduced by phylogenetic analysis [61]. 

The conserved epitopes allow generating immunity that is not only cross-protective over coronaviruses but also relatively resistant to ongoing virus evolution as well as future pandemics. Consequently, in order to make the immunizing activity of vaccines independent from the appearance of S-protein variants, a road map must be drawn using a platform based on the use of cryptic specific synthetic peptides to identify an epitope-specific vaccine [25]. The amino acid sequences of the oligopeptides reflect the epitopes belonging to the cryptic zone of S protein, which cannot be mutated under penalty of non-functionality of the protein itself.

At last, the use of oligopeptides allows the possibility of producing large quantities of vaccine at relatively low cost and easy handling in the transport and storage chain and methods of administration. Moreover, this strategy allows us to obtain a polyclonal antiserum where it is monoclonal for FABs and polyclonal for FC on which all the reactions for the T-cell-mediated response then depend.

Methods to generate specific antisera against peptide are widely known; antibodies are produced by the repeated immunization of inbred animals (mice, rats, rabbits, etc.). 

The use of simple antigenic formulations compared to whole virus-based vaccine requires carriers or adjuvants to enhance vaccine immunogenicity. Among the large variety of nanoparticles type, gold nanoparticles (AuNPs) have raised a great deal of excitement because of their good biocompatibility, low toxicity, stability, and small dimension [62]. Importantly, they are useful for therapeutic application because they are apt to functionalization with antigens, and in fact, they increase the antigen presentation process [63] by promoting the effective maturation of dendritic cells and the proliferation of Th and NK cells accompanied by an increased secretion of cytokines.

We believe that the peptide vaccination platform described here offers unique advantages over other candidate vaccines, such as rapid manufacturing in response to sequence mutations (compared to protein-based or viral-vector-based vaccines), and greater stability at room temperature (compared to RNA-based vaccines). With an increasing number of people having been immunized against SARS-CoV-2 with an RNA-, adenovirus-, or protein-based vaccine, COVID-eVax might be also considered as an additional platform for booster immunizations to extend the duration of protective immunity. In future scenarios, the use of highly conserved epitopes (short sequences of amino acids) of a protein antigen belonging to a pandemic agent represents an excellent strategy for the preparation of active vaccines even in the presence of future functional variants.

Another strategy, highly regarded by us, could be the use of peptides to prevent virus entry into host cells. It was shown that DPP4 acts as a SARS-CoV co-receptor, thus suggesting a potential similar mechanism of entry for SARS-CoV-2 [64]. Specifically, residues of the S1 domain are predicted to interact with both ACE2 and DPP4 [65]. 

In COVID-19, the dynamic of correlation between DPP4/CD26 localization and site of lung inflammation appeared to be confirmed [66]. Recent data clearly indicated that the aggressive impact of CoVs on tissues and organs is preferentially modulated, or least co-modulated, by DPP4 [67], and that DPP4 inhibition could antagonize this mechanism. Thus, the use of DPP4 peptide may represent an innovative approach to be employed for the pharmacologic treatment of COVID-19. Computational studies revealed that among the two DPP4-derived synthetic peptides, DPP4_270–295_ showed a higher binding affinity toward RBD with respect to DPP4_318–343_ driven by its higher vdW contribution, which is both dominant and proportional at the RBD interface. Indeed, our results demonstrate that DPP4_270–295_ acts in order to mask virus to intercept the DPP4 receptor and other known receptors on target cells, inhibiting the virus entry. The efficacy of using two peptides in combination was higher than that obtained with a single peptide alone induced by increased greed in the binding reaction.

- Thus, our results indicate the potential use of the peptide platform for the production of small peptides able to inhibit early virus entry into host cells.
- On the other side, this platform allows producing specific antibodies (polyclonal and monoclonal) against the S protein of SARS-CoV-2 [68,69]. In particular, using peptides focused on the conserved RBD region, the antibody produced will be effective for preventing current and future variants that ineluctably will arise during pandemics.
- Future in vivo experiments will be performed to immunize transgenic animals (human ACE2 protein expression in the lung) or hamster models [70] before the administration of isovirus carrying the known different variants. Animal experiments make it possible to identify the dose, timing, and method of administration of the vaccine and also to evaluate any unwanted pathological effects.

## Figures and Tables

**Table 1:** Selected peptides sequences utilized.

	*Position*	*Sequence*
ACE2	21–45	IEEQAKTFLDKFNHEAEDLFYQS
DPP4	270–295	VVNTDSLSSVTNATSIQITAPASMLI
	318–343	RIQNYSVMDICDYDESSGRWNCLVAR
SPIKE		
1	484–508	EGFNCYFPLQSYGFQPTNGVGYQPY
2	453–476	YRLFRKSNLKPFERDISTEIYQAGS
3	402–427	IRGDEVRQIAPGQTGKIADYNYK
4	322–341	PTESIVRFPNIFNATRFASV

**Table 2:** Comparison between the cluster statistics of DPP4_270–295_ and DPP4_318–343_ after molecular recognition with RBD-up conformation through the HADDOCK tool.

	HADDOCK Score *	Cluster Size	RMSD **	VdW Energy	Electrostatic Energy	Desolvation Energy	BSA ***	Z-Score ****
DPP4_270-295_	−98.1 +/− 2.9	53	0.2 +/− 0.2	−59.6 +/− 2.8	−108.5 +/− 15.9	−17.8 +/− 2.8	1441.4 +/− 68.1	−2.5
DPP4_318-343_	−90.5 +/− 4.2	10	2.2 +/− 0.1	−48.4 +/− 8.5	−127.4 +/− 16.5	−18.1 +/− 2.6	1270.0 +/− 23.4	−1.3

* The HADDOCK score is defined as: 1.0 Evdw + 0.2 Eelec + 1.0 Edesol + 0.1 EAIR. ** Root Main Square Deviations (RMSD) from the overall lowest-energy structure. *** Buried Surface Area. **** HADDOCK Z-score indicates standard deviations from the average cluster (the more negative, the better).

**Table 3:** Binding affinity of DPP4_270–295_ and DPP4_318–343_ in complex with S protein and their dissociation constant (K_D_) at 37  °C.

	DPP4_270–295_		DPP4_318–343_	
Temperature (°C)	Binding affinity ΔG (kcal mol^−1^)	Dissociation constant Kd (M)	Binding affinity ΔG (kcal mol^−1^)	Dissociation constant Kd (M)
37°	−11.8	4.5 × 10^−9^	−9.0	4.8 × 10^−7^

**Table 4:** Comparison between the cluster statistics of DPP4_270–295_ in complex with B1.1.7, B1.351, and P.1 mutated complexes, after molecular recognition through the HADDOCK tool.

	HADDOCK Score *	Cluster Size	RMSD **	VdW Energy	Electrostatic Energy	Desolvation Energy	BSA ***	Z-Score ****
B.1.1.7 DPP4_270–295_	−89.5 +/− 1.9	24	0.4 +/− 0.2	−55.7 +/− 3.0	−94.8 +/− 12.1	−15.1 +/− 2.2	1424.0 +/− 107.7	−2.4
B.1.351 DPP4_270–295_	−84.3 +/− 5.6	26	2.2 +/− 0.0	−50.7 +/− 7.7	−85.2 +/− 18.5	−18.9 +/− 2.5	1338.5 +/− 69.0	−2.2
P.1 DPP4_270–295_	−95.3 +/− 2.4	52	0.4 +/− 0.2	−62.0 +/− 2.1	−115.8 +/− 26.7	−10.3 +/− 2.3	1485.9 +/− 65.5	−2.6

**Table 5:** BALB/c MHC class I epitopes in predicted models.

Allele	Spike Sequences	Start	End	Length	Peptide	Predicted IC50	Rank
H-2-Dd	1	6	14	9	YFPLQSYGF	0.000033	0.8
H-2-Dd	1	1	9	9	EGFNCYFPL	0.000035	0.8
H-2-Kd	2	1	9	9	YRLFRKSNL	0.000042	1.6
H-2-Kd	1	7	15	9	FPLQSYGFQ	0.000050	1.9
H-2-Kd	4	11	19	9	IFNATRFAS	0.000065	2.3
H-2-Dd	4	7	15	9	RFPNIFNAT	0.000104	2.6
H-2-Kd	2	12	20	9	FERDISTEI	0.000121	3.8
H-2-Dd	4	12	20	9	FNATRFASV	0.000129	3.2
H-2-Kd	1	5	13	9	CYFPLQSYG	0.000134	4.2
H-2-Kd	1	11	19	9	SYGFQPTNG	0.000158	4.9
H-2-Kd	1	12	20	9	YGFQPTNGV	0.000197	6
H-2-Dd	1	12	20	9	YGFQPTNGV	0.000206	5
H-2-Dd	4	11	19	9	IFNATRFAS	0.000300	7.2
H-2-Dd	2	1	9	9	YRLFRKSNL	0.000309	7.4
H-2-Dd	4	2	10	9	TESIVRFPN	0.000342	8
H-2-Dd	2	4	12	9	FRKSNLKPF	0.000382	8.7
H-2-Dd	1	14	22	9	FQPTNGVGY	0.000425	9.7
H-2-Dd	3	14	22	9	TGKIADYNY	0.000511	13
H-2-Dd	4	9	17	9	PNIFNATRF	0.000542	14
H-2-Dd	3	9	17	9	IAPGQTGKI	0.000555	14
H-2-Kd	2	7	15	9	SNLKPFERD	0.000578	16
H-2-Dd	4	3	11	9	ESIVRFPNI	0.000608	15
H-2-Kd	3	15	23	9	GKIADYNYK	0.000613	16
H-2-Dd	3	11	19	9	PGQTGKIAD	0.000691	17
H-2-Dd	4	6	14	9	VRFPNIFNA	0.000703	17
H-2-Dd	3	3	11	9	GDEVRQIAP	0.000725	17
H-2-Dd	2	9	17	9	LKPFERDIS	0.000740	18
H-2-Dd	1	8	16	9	PLQSYGFQP	0.000745	18
H-2-Dd	2	2	10	9	RLFRKSNLK	0.000849	20
H-2-Dd	1	9	17	9	LQSYGFQPT	0.000992	22
H-2-Dd	2	10	18	9	KPFERDIST	0.001060	23
H-2-Kd	2	8	16	9	NLKPFERDI	0.001126	25
H-2-Kd	4	2	10	9	TESIVRFPN	0.001152	25
H-2-Dd	2	16	24	9	ISTEIYQAG	0.001178	25
H-2-Dd	2	6	14	9	KSNLKPFER	0.001218	25
H-2-Kd	4	7	15	9	RFPNIFNAT	0.001245	26
H-2-Kd	3	8	16	9	QIAPGQTGK	0.001282	27
H-2-Dd	3	6	14	9	VRQIAPGQT	0.001547	30
H-2-Kd	3	7	15	9	RQIAPGQTG	0.001553	30
H-2-Dd	1	4	12	9	NCYFPLQSY	0.001557	30
H-2-Dd	3	2	10	9	RGDEVRQIA	0.001560	30
H-2-Dd	4	10	18	9	NIFNATRFA	0.001674	31
H-2-Dd	1	7	15	9	FPLQSYGFQ	0.001885	34
H-2-Dd	1	2	10	9	GFNCYFPLQ	0.002278	38
H-2-Dd	4	5	13	9	IVRFPNIFN	0.002596	41
H-2-Kd	4	4	12	9	SIVRFPNIF	0.002697	41
H-2-Kd	3	1	9	9	IRGDEVRQI	0.002699	41
H-2-Kd	2	3	11	9	LFRKSNLKP	0.002716	41
H-2-Kd	4	12	20	9	FNATRFASV	0.002880	43
H-2-Kd	2	4	12	9	FRKSNLKPF	0.002885	43
H-2-Kd	3	2	10	9	RGDEVRQIA	0.002892	43
H-2-Dd	2	13	21	9	ERDISTEIY	0.002938	44
H-2-Kd	1	9	17	9	LQSYGFQPT	0.003065	44
H-2-Dd	1	17	25	9	TNGVGYQPY	0.003155	46
H-2-Dd	2	12	20	9	FERDISTEI	0.003191	47
H-2-Kd	1	6	14	9	YFPLQSYGF	0.003251	46
H-2-Dd	4	4	12	9	SIVRFPNIF	0.003263	47
H-2-Kd	4	9	17	9	PNIFNATRF	0.003361	46
H-2-Dd	2	17	25	9	STEIYQAGS	0.003431	49
H-2-Kd	3	9	17	9	IAPGQTGKI	0.003609	48
H-2-Kd	2	16	24	9	ISTEIYQAG	0.003758	49
H-2-Kd	1	1	9	9	EGFNCYFPL	0.004132	51
H-2-Dd	4	1	9	9	PTESIVRFP	0.004211	55
H-2-Kd	2	11	19	9	PFERDISTE	0.004236	52
H-2-Dd	4	8	16	9	FPNIFNATR	0.004272	55
H-2-Dd	3	1	9	9	IRGDEVRQI	0.004426	56
H-2-Kd	1	13	21	9	GFQPTNGVG	0.004526	53
H-2-Kd	4	3	11	9	ESIVRFPNI	0.004574	53
H-2-Dd	1	3	11	9	FNCYFPLQS	0.004609	57
H-2-Kd	1	17	25	9	TNGVGYQPY	0.004972	55
H-2-Kd	4	10	18	9	NIFNATRFA	0.004999	55
H-2-Dd	2	5	13	9	RKSNLKPFE	0.005308	62
H-2-Kd	2	17	25	9	STEIYQAGS	0.006395	61
H-2-Kd	4	6	14	9	VRFPNIFNA	0.006504	62
H-2-Kd	1	14	22	9	FQPTNGVGY	0.006524	62
H-2-Dd	3	15	23	9	GKIADYNYK	0.006682	69
H-2-Kd	4	8	16	9	FPNIFNATR	0.006707	62
H-2-Kd	3	11	19	9	PGQTGKIAD	0.006785	63
H-2-Kd	2	2	10	9	RLFRKSNLK	0.006786	63
H-2-Dd	3	7	15	9	RQIAPGQTG	0.007514	72
H-2-Dd	3	4	12	9	DEVRQIAPG	0.007684	73
H-2-Kd	1	15	23	9	QPTNGVGYQ	0.007849	66
H-2-Dd	3	5	13	9	EVRQIAPGQ	0.007858	73
H-2-Kd	2	13	21	9	ERDISTEIY	0.007966	66
H-2-Kd	3	6	14	9	VRQIAPGQT	0.008057	66
H-2-Dd	3	13	21	9	QTGKIADYN	0.008880	77
H-2-Dd	2	15	23	9	DISTEIYQA	0.008993	77
H-2-Dd	1	15	23	9	QPTNGVGYQ	0.009217	78
H-2-Kd	2	14	22	9	RDISTEIYQ	0.009264	70
H-2-Kd	3	10	18	9	APGQTGKIA	0.009298	70
H-2-Kd	2	5	13	9	RKSNLKPFE	0.009471	70
H-2-Dd	1	11	19	9	SYGFQPTNG	0.009661	79
H-2-Dd	2	7	15	9	SNLKPFERD	0.010219	81
H-2-Kd	2	9	17	9	LKPFERDIS	0.010252	72
H-2-Dd	2	14	22	9	RDISTEIYQ	0.011202	83
H-2-Dd	2	11	19	9	PFERDISTE	0.011614	84
H-2-Kd	3	3	11	9	GDEVRQIAP	0.012688	77
H-2-Kd	1	4	12	9	NCYFPLQSY	0.012694	77
H-2-Kd	3	4	12	9	DEVRQIAPG	0.012768	77
H-2-Kd	1	3	11	9	FNCYFPLQS	0.013268	78
H-2-Dd	3	8	16	9	QIAPGQTGK	0.013684	88
H-2-Dd	1	10	18	9	QSYGFQPTN	0.013791	88
H-2-Kd	3	14	22	9	TGKIADYNY	0.016227	82
H-2-Kd	3	12	20	9	GQTGKIADY	0.016425	83
H-2-Dd	1	16	24	9	PTNGVGYQP	0.016657	91
H-2-Kd	4	1	9	9	PTESIVRFP	0.017077	84
H-2-Dd	3	12	20	9	GQTGKIADY	0.018247	93
H-2-Kd	3	5	13	9	EVRQIAPGQ	0.019432	86
H-2-Dd	3	10	18	9	APGQTGKIA	0.019829	94
H-2-Kd	1	16	24	9	PTNGVGYQP	0.020156	87
H-2-Dd	1	5	13	9	CYFPLQSYG	0.020267	94
H-2-Dd	2	3	11	9	LFRKSNLKP	0.022977	96
H-2-Kd	4	5	13	9	IVRFPNIFN	0.023020	89
H-2-Kd	3	13	21	9	QTGKIADYN	0.023357	90
H-2-Kd	2	6	14	9	KSNLKPFER	0.026144	92
H-2-Kd	1	2	10	9	GFNCYFPLQ	0.026266	92
H-2-Kd	2	10	18	9	KPFERDIST	0.027065	92
H-2-Kd	2	15	23	9	DISTEIYQA	0.027473	92
H-2-Kd	1	8	16	9	PLQSYGFQP	0.027964	93
H-2-Dd	2	8	16	9	NLKPFERDI	0.028100	98
H-2-Dd	1	13	21	9	GFQPTNGVG	0.028708	98
H-2-Kd	1	10	18	9	QSYGFQPTN	0.040577	97

**Table 6:** Prediction of antigenic processing and presentation for potential T-cell epitopes of spike peptides.

Supertype	Peptide	Binding Affinity	Rescale Binding Affinity	Proteosomal C-Terminal Cleavage	Transport Affinity	Prediction Score	MHC-I Binding
A1	FQPTNGVGY	0.1117	0.4741	0.7859	2.8600	0.7583	< -E
A1	ERDISTEIY	0.2097	0.8903	0.9734	2.6460	1.1686	< -E
A26	FQPTNGVGY	0.2611	0.7007	0.9409	2.8600	0.9848	< -E
